# Neural Radiance Field Dynamic Scene SLAM Based on Ray Segmentation and Bundle Adjustment

**DOI:** 10.3390/s25061679

**Published:** 2025-03-08

**Authors:** Yuquan Zhang, Guosheng Feng

**Affiliations:** 1 School of Traffic and Transportation, Shijiazhuang Tiedao University, Shijiazhuang 050043, China; yqzhang_qc@163.com; 2 Department of Automotive Engineering, Hebei Jiaotong Vocational and Tecenical College, Shijiazhuang 050035, China; 3 School of New Energy Vehicle Engineering, Guangzhou Institute of Science and Technology, Guangzhou 510540, China

**Keywords:** Dense SLAM, neural implicit coding, surface rendering

## Abstract

The current neural implicit SLAM methods have demonstrated excellent performance in reconstructing ideal static 3D scenes. However, it remains a significant challenge for these methods to handle real scenes with drastic changes in lighting conditions and dynamic environments. This paper proposes a neural implicit SLAM method that effectively deals with dynamic scenes. We employ a keyframe selection and tracking switching approach based on Lucas–Kanade (LK) optical flow, which serves as prior construction for the Conditional Random Fields potential function. This forms a semantic-based joint estimation method for dynamic and static pixels and constructs corresponding loss functions to impose constraints on dynamic scenes. We conduct experiments on various dynamic and challenging scene datasets, including TUM RGB-D, Openloris, and Bonn. The results demonstrate that our method significantly outperforms existing neural implicit SLAM systems in terms of reconstruction quality and tracking accuracy.

## 1. Introduction

Throughout the years, Simultaneous Localization and Mapping (SLAM) [1,2,3,4,5,6,7,8] has been regarded as a promising solution for addressing the challenges in AR/VR [9] and autonomous driving technologies [10]. Compared to sparse SLAM systems, SLAM systems with dense visual information [11] have broader application prospects. Traditional dense SLAM systems are capable of building high-fidelity maps, but they have limitations in terms of texture details and hole filling. Recently, the outstanding performance exhibited by Neural Radiance Fields [12] has garnered widespread attention. By learning shape priors through neural networks and embedding input point coordinates into a high-dimensional space, the neural-based network achieves high-fidelity geometry reconstruction, preserving more high-frequency details. Consequently, SLAM methods based on Neural Radiance Fields (NeRFs) [13,14,15,16] have emerged.

Compared to traditional dense SLAM methods, neural implicit methods have shown good performance in location scene completion, background restoration, memory consumption, and obtaining better anomaly handling from shape priors. As a pioneering work, iMAP [17] can update scenes in real time and update unknown areas. NICE-SLAM [18] employs a multi-resolution feature grid to avoid oversmoothing and the forgetting problem of a single MLP, enabling the storage and representation of large-scale scenes. ESLAM [19] achieves faster and more accurate reconstruction by using multi-scale axis-aligned feature planes. Co-SLAM [20] proposes a joint coordinate and sparse grid-encoding method that balances optimization speed and geometric accuracy. However, in real-world scenarios, there often exist interferences caused by moving people/objects. Up to now, existing NeRF-based SLAM methods have not come up with effective solutions. When facing dynamic scenes, existing methods will have significant tracking and mapping errors. Although NICE-SLAM has proposed a method for handling dynamic objects, it cannot accomplish effective tracking and reconstruction in highly dynamic or heavily occluded dynamic scenes.

Currently, neural implicit SLAM systems typically assume uniform motion for tracking, and this assumption is broken in dynamic environments, resulting in tracking errors. Moreover, pixel losses caused by dynamic objects also affect the quality of mapping. Compared to NICE-SLAM’s method of removing dynamic pixels by controlling pixel thresholds, we extract feature points to construct inter-frame optical flow vectors, calculate inter-frame displacement and reprojection errors, and build a unary potential function. We introduce loop closure detection based on inter-frame matching. By adopting semantic detection based on YOLOv8 and using pixel color correlations and reprojection errors to construct a binary potential function, we segment dynamic pixels in the scene and construct the corresponding loss function, achieving dynamic object removal and scene reconstruction.

We propose three contributions:

- We propose a joint dynamic pixel detection and segmentation method based on semantic segmentation and Conditional Random Fields (CRFs), adaptively adjusting the loss function to achieve tracking and mapping in dynamic environments. By maintaining a set of dynamic pixels, we accomplish pixel filling and completion.
- We propose a semantic-based neural implicit dynamic SLAM framework that utilizes an Implicit Truncated Signed Distance Field (TSDF) representation, enabling tracking and mapping in dynamic environments.
- We propose the loop closure detection and keyframe selection strategies based on Lucas–Kanade (LK) optical flow, accomplishing inter-frame matching by calculating optical flow vectors. We incorporate loop closure keyframes into the optimization process, implementing loop closure detection in the neural implicit SLAM system.

## 2. Related Work

### 2.1. Dynamic Visual SLAM

Simultaneous Localization and Mapping (SLAM) can be divided into laser SLAM, visual SLAM, and multi-sensor fusion SLAM systems based on different sensors. Among them, visual SLAM has shown wide adaptability in both virtual and real scenes. However, traditional visual SLAM systems often lack robustness when dealing with dynamic objects in the real world, such as changing viewpoints, lighting conditions, and scenes. In recent years, many researchers have proposed solutions to the SLAM problem in dynamic environments. DS-SLAM [21] uses an independent semantic thread to segment the dynamic parts of the scene. DynaSLAM [22] utilizes a CNN-based semantic prior system for pixel segmentation and combines multi-view geometry to remove features of dynamic objects. DynaSLAM II [23] tightly couples the scene structure, camera poses, and dynamic object trajectories within the same optimization window. LC-CRF SLAM [24] utilizes dynamic feature partitioning methods based on graph cuts and Conditional Random Fields to segment dynamic pixels in the scene. DRG-SLAM [25] proposes a dynamic feature extraction method that combines semantic segmentation and epipolar constraints, and it also improves the robustness of the SLAM system in weakly textured and dynamic environments using point-line features. However, these methods are all based on traditional SLAM systems and cannot effectively infer the unobserved parts of the scene or fill scene holes. The rendering quality of implicitly represented scenes is not as realistic as shape and texture details compared to explicit representation.

### 2.2. Neural Implicit SLAM

Current neural implicit SLAM methods have achieved many results. For example, iMAP [17] proposes a real-time dense SLAM system that can fill unknown areas but cannot handle inference in large-scale scenes. NICE-SLAM [18] builds upon iMAP and proposes a system based on a multi-resolution feature grid. It uses pretrained multiple MLP decoders to decode different occupancy values and introduces a dynamic object removal method based on pixel loss. It conducts experiments on the Co-fusion dataset [26] but cannot be applied to highly dynamic scenes and residual artifacts remain after removing moving objects’ effects. ESLAM [19] utilizes a multi-scale axis-aligned TSDF for geometric representation and achieves better geometric feature expression. NeRF-SLAM [27] combines the frontend of Droid-SLAM [28] and the encoding of instant-ngp to achieve real-time single-view RGB reconstruction. Co-SLAM [20] combines sparse grid encoding and prior coordinate encoding to balance optimization speed and local details. However, in dynamic scenes, Co-SLAM exhibits significant drift in tracking, and the tracking accuracy is greatly affected in real scenes with a large viewpoint and lighting changes. The main challenges in dynamic scenes arise from occlusion caused by dynamic objects, and existing systems suffer from severe drift when there are large changes in viewpoint. We believe that in the presence of dynamic objects in the scene, semantic segmentation should be introduced to divide dynamic and static pixels, and light sampling should be readjusted. Additionally, to enhance tracking robustness, feature-point-based LK optical flow should be introduced to enable loop detection and more efficient bundle adjustment processes.

## 3. Methodology

Traditional semantic-based SLAM systems, such as DynaSLAM [22] based on Mask R-CNN, can achieve the pixel-level segmentation of potential dynamic targets and background information but often struggle to meet the real-time performance requirements of SLAM. Furthermore, traditional methods exhibit significant shortcomings in mapping accuracy and the acquisition of high-fidelity models. To improve efficiency and reduce computational costs, this paper proposes a SLAM framework based on deep learning, ray segmentation, and optical flow loop closure detection. The system adopts a lightweight, single-stage object detection method based on YOLOv8 to identify dynamic objects within the scene. To accurately utilize semantic masks for reconstruction and improve reconstruction quality, a ray-removal-based reconstruction strategy is employed, which directly excludes dynamic rays from the reconstruction process, significantly reducing reconstruction artifacts and enhancing tracking accuracy.

Additionally, when object detection boxes cover large areas of the background in the image, directly removing all feature points within the detection boxes may weaken geometric constraints in pose estimation. To address this, the paper introduces a method combining object detection with depth information to achieve foreground and background segmentation. Based on image segmentation, dynamic features are efficiently identified through optical flow hypothesis testing. Finally, using the static optical flow formed by the obtained sparse feature points, the system proposes an optical flow scoring strategy to effectively perform loop closure detection and global Bundle Adjustment (BA) optimization.

**Figure 1:**
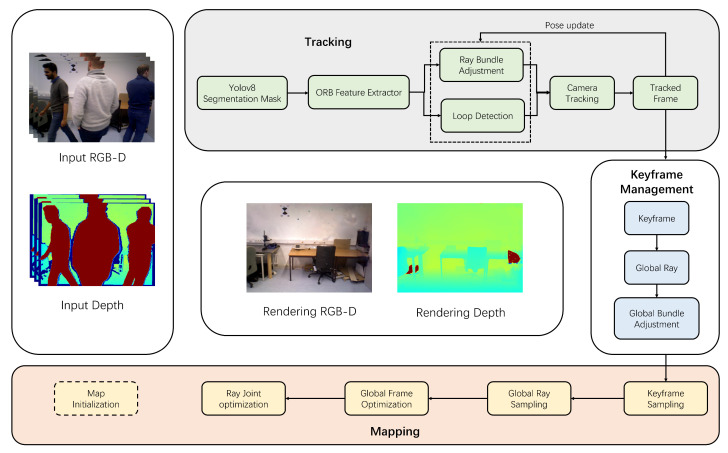
The overall framework of our method. Our system architecture is divided into two main threads, including tracking and mapping, which are fused and connected through a keyframe management module. We utilize a mask module based on YoloV8 to obtain dynamic masks, perform dynamic light removal, correct the camera pose, and transmit the keyframes into the keyframe management module. In the mapping thread, filtered static lights are shared and utilized for Neural Radiance Field (NeRF) reconstruction. When loop closure is detected, global loop closure adjustment is simultaneously triggered in both tracking and mapping, ensuring that our tracking and reconstruction processes can be corrected in unison.

The architecture of the dynamic environment SLAM system based on Neural Radiance Fields is shown in Figure 1. The system first receives input data from an RGB-D sensor, including color images and depth information, for subsequent processing. In the tracking thread, the system samples *N* rays from each pixel and performs semantic segmentation using YOLOv8 to identify dynamic and static objects. Dynamic object rays are then marked to ensure that dynamic pixels do not affect results during photometric error minimization updates. Subsequently, rays associated with dynamic objects are removed, and bundle adjustment is performed.

Simultaneously, the mapping thread extracts feature points based on the initial pose provided by the tracking thread and marks dynamic feature points using semantic segmentation results from YOLOv8. After removing these feature points, the system constructs sparse optical flow and optimizes the keyframes of static scenes. Loop closure detection is performed between keyframes to identify similar frames, triggering bundle adjustment in loop frames to optimize camera poses. Finally, the system learns the rays of the scene using a MLP to generate volumetric density representations, and through multi-view rendering, the rays are synthesized into voxel representations. This results in the generation of a mesh model of the scene, completing the 3D reconstruction of the dynamic environment.

### 3.1. Dynamic and Static Pixel Segmentation Based on Conditional Random Fields

Although current neural implicit SLAM methods can handle low-dynamic environments to a certain extent, they still face significant challenges in highly dynamic and complex environments, particularly those with moving occlusions, which can greatly impact the quality of the generated mesh. Furthermore, as most neural implicit SLAM systems currently employ direct methods that track by minimizing photometric errors through iterative ray optimization, this paper proposes removing dynamic rays while constructing a Conditional Random Fields for refined removal.

Initially, dynamic pixels $IDP_{d}$ and the corresponding dynamic field range *M* are obtained by applying YOLOv8 to the input RGB image. Additionally, a mask is generated for cropped 40×40 image patches, using their boundary ranges *M* and center positions, which incorporates a mixture of dynamic and static information along the edges.

To obtain optimal dynamic object masks and static scene masks, Conditional Random Fields are utilized to filter the edges of the dynamic masks. The pairwise potential function is defined as follows:

(1) $$W\left(y_{i},y_{j}\right)=\alpha_{0}Sim\left(x_{i},x_{j}\right)+\beta_{0}Spatial\left(x_{i},x_{j}\right)$$

where $x_{i}$ and $x_{j}$ represent the features of two different image pixels, and $y_{i}$ and $y_{j}$ are their corresponding labels. The similarity between $x_{i}$ and $x_{j}$ is measured by the similarity function $Sim(x_{i},x_{j})$, while $Spatial(x_{i},x_{j})$ describes the spatial relationship between these pixels. Here, $\alpha_{0}$ and $\beta_{0}$ are introduced as weighting coefficients for the similarity function and the spatial relationship function, respectively. Specifically, $\alpha_{0}$ controls the contribution of the similarity function $Sim(x_{i},x_{j})$, and $\beta_{0}$ determines the influence of the spatial relationship function $Spatial(x_{i},x_{j})$ in the overall computation. Similar to NICE-SLAM, we sample pixels $m_{i}$ and $n_{i}$ along the light rays, and the relationship between them is computed using the Pearson correlation coefficient, which is defined by the following:

(2) $$r=\frac{\mathrm{cov}(X_{1},X_{2})}{\sigma \left(X_{1}\right)\sigma \left(X_{2}\right)}$$

where $\mathrm{cov}(X_{1},X_{2})$ represents the covariance between the normalized pixel values $m_{i}$ and $n_{i}$, and $\sigma (X_{1})$ and $\sigma (X_{2})$ denote the standard deviations of the normalized pixel values $m_{i}$ and $n_{i}$, respectively. Pixels with a correlation coefficient below *k* (initially set to 0.5) are classified as dynamic pixels. Due to projection errors in dynamic pixels being significantly larger, the potential function $Spatical(x_{i},x_{j})$ is applied to these pixels. For the *i*-th pixel, assume the corresponding projection error is $e_{i}$. Considering that the dynamic scene includes all dynamic pixels within it, the weight $\lambda_{z}$ increases as pixels are further away from the center *M* of the dynamic scene. The weight $\lambda_{z}$ is calculated as shown below:

(3) $$\lambda_{z}=1/\left(1+\alpha_{z}d\right)$$

where $\alpha_{z}$ is initially set to 0.5. The weighted average of all pixel projection errors is computed to obtain the threshold value *t*. The calculation formula is shown below:

(4) $$t=\frac{\sum \left(e_{i}w_{i}\right)}{\sum w_{i}}$$

where $e_{i}$ represents the projection error of the *i*-th pixel, and $w_{i}$ represents the weight of the *i*-th pixel. The corresponding feature function is thus calculated as shown below:

(5) $$f_{k}\left(x_{i},y_{i}\right)=Sim\left(x_{i},x_{i}\right)<k,Spatial\left(x_{i},x_{i}\right)<t$$

By utilizing Conditional Random Fields, the optimal dynamic object label map $P_{m}^{d}$ and the corresponding pixel depth map $P_{m}^{s}$ are obtained. To balance the dynamic processing of different scenes, different parameters are set to distinguish between low-dynamic and high-dynamic scenes. By using the ratio of dynamic to static parts, denoted as $W_{\mathrm{ratio}}=P_{m}^{d}/P_{m}^{s}$, when the value of *W* in a scene is less than 0.1, it is considered a low-dynamic scene. Conversely, when *W* is greater than 0.1, it is considered a high-dynamic scene.

Compared to traditional methods for removing low-dynamic pixels, this method effectively filters all sampling rays. Traditional methods usually passively remove sampled pixels *M* through displacement maps, whereas this method directly operates on sampled rays of specific pixels in the neural implicit representation. This allows direct adjustment during the rendering process, thereby reducing redundant computational steps while achieving precise rendering. The corresponding boundary depth $B_{\mathrm{depth}}$ of the rendering rays is obtained through an iterative sampling process.

The boundary is defined to exclude rays outside the specified area and to remove dynamic pixel depth maps $D_{m}^{d}$ and rays within the scene from being rendered. The corresponding depth value is set to 0, and the nearest neighbor search is conducted to fill in the missing edges. To account for edge pixel loss, the number of neighboring pixels for ray sampling is increased from 16 to 32. Under these specific ratios, the depth losses of static objects and dynamic objects are calculated separately, and the color loss $L_{\mathrm{color}}$ is also computed if the pixel is not fully rendered. Finally, the static depth loss $L_{\mathrm{static}}$ and the dynamic depth loss $L_{\mathrm{dynamic}}$ are determined to calculate the total loss $L_{\mathrm{total}}$.

(6) $$L_{\mathrm{static}}=\sum M_{\mathrm{static}}\left|\frac{B_{\mathrm{depth}}-C_{\mathrm{color}}}{\sqrt{\mathrm{uncertainty}+1\times 10^{-10}}}\right|$$

(7) $$L_{\mathrm{dynamic}}=\sum M_{\mathrm{dynamic}}\left|\frac{B_{\mathrm{depth}}-C_{\mathrm{color}}}{\sqrt{\mathrm{uncertainty}+1\times 10^{-10}}}\right|$$

(8) $$L_{\mathrm{total}}=w_{\mathrm{static}}*L_{\mathrm{static}}+w_{\mathrm{dynamic}}*L_{\mathrm{dynamic}}$$

Following this virtual trajectory, the parameters of the Gaussian smoothing filter are adjusted to achieve a better estimation of the target position. By initializing $w_{\mathrm{dynamic}}$ and $w_{\mathrm{static}}$ to 0.5, adjustments are avoided in areas with color loss, thereby preserving image quality.

### 3.2. Neural Implicit Rendering for Dynamic Environments

We adopt a hierarchical feature grid with coarse and fine levels for scene representation. The coarse grid is used to represent low-frequency or textureless areas, while the fine grid captures high-frequency details. Subsequently, grids with edge lengths of 16 cm and 4 cm are used to hierarchically process the scene. The features from the grids are fed into an MLP, where the parameters are set to 64 and 3×64, respectively. This process generates the coarse-level signed distance function (SDF) values ($s^{\mathrm{coarse}}$) and their features ($z^{\mathrm{coarse}}$) as well as the fine-level SDF values ($s^{\mathrm{fine}}$) and their features ($z^{\mathrm{fine}}$).

(9) $$s^{\mathrm{coarse}},z^{\mathrm{coarse}}=f^{\mathrm{coarse}}\left(\gamma \left(x\right),\Phi^{\mathrm{coarse}}\left(x\right)\right)$$

(10) $$\Delta s,z^{\mathrm{fine}}=f^{\mathrm{fine}}\left(\gamma \left(x\right),\Phi^{\mathrm{fine}}\left(x\right)\right)$$

$\hat{n}$ denotes the normal vector, *v* represents the viewing direction, and $\Phi_{\mathrm{color}}\left(x\right)$ indicates the result of trilinear interpolation for color features at point *x*. To clarify, γ corresponds to the fixed positional encoding, and Φ(x) represents the result of trilinear interpolation on the feature grid at point *x*. When the base SDF values from the coarse level and the final residual SDFδ are given, the final predicted SDF value $\hat{s}$ at point *x* is the sum of these two components:

(11) $$\hat{s}=s^{\mathrm{coarse}}+\Delta s$$

To further optimize distance calculations and surface reconstruction, we introduce the Truncated Signed Distance Function (TSDF). TSDF allows for more precise surface representation and truncates distance values to a certain threshold, enhancing the stability of depth information. Specifically, TSDF is computed by truncating the SDF values to a predefined range, thereby preventing distant regions from influencing the final rendering result excessively.

To compute TSDF, we first calculate the signed distance values for each point on the coarse and fine grids, and then we apply truncation:

(12) TSDF(x)=sign(s(x))·min(|s(x)|,T)

where *T* is a predefined truncation threshold, and s(x) represents the signed distance function value at point *x*. We then combine the fine-level SDF and the coarse-level TSDF values to obtain a more accurate surface description.

By incorporating TSDF, we significantly improve the representation of surfaces, particularly in dynamic environments with moving objects. TSDF enables more stable depth and surface information, even in challenging scenes.

Similarly, color rendering can be computed as follows:

(13) $$\hat{c}=f^{\mathrm{color}}\left(x,\hat{n},\gamma \left(v\right),z^{\mathrm{coarse}},z^{\mathrm{fine}},\Phi^{\mathrm{color}}\left(x\right)\right)$$

We assume a ray *r* is emitted from the camera center *o* along the normalized viewing direction *v* of a pixel. Subsequently, *N* points are sampled along the ray with their predicted SDF values and color values denoted as ${\hat{s}}_{i}$ and ${\hat{c}}_{i}$, respectively.

(14) $$\hat{C}=\sum_{i=1}^{N}T_{i}\alpha_{i}{\hat{c}}_{i}$$

(15) $$T_{i}=\prod_{j=1}^{i-1}\left(1-\alpha_{j}\right)$$

(16) $$\alpha_{i}=1-e^{-\sigma_{i}\delta_{i}}$$

where $T_{i}$ corresponds to the opacity of the sampled point *i* on the ray *r*, while $\delta_{i}$ represents the distance between adjacent sampled points. Similarly, the depth $\hat{D}$ of the surface intersecting with the current ray *r* can also be computed:

(17) $$\hat{D}=\sum_{i=1}^{N}T_{i}\alpha_{i}t_{i}$$

Finally, the color and depth rendering losses, $L_{rgb}$ and $L_{d}$, are computed as the Euclidean distances between the rendered results and the observed results, as expressed in the following formulas:

(18) $$L_{rgb}=\frac{1}{N}\sum_{n=1}^{N}{\left({\hat{c}}_{n}-c_{n}\right)}^{2}$$

(19) $$L_{d}=\frac{1}{\left|R_{d}\right|}\sum_{r\in R_{d}}{\left({\hat{d}}_{r}-D\left(u,v\right)\right)}^{2}$$

### 3.3. Optical Flow-Based Dynamic Tracking and Loop Closure Detection

Current neural implicit SLAM methods often exhibit significant tracking errors in challenging environments with large photometric variations. To enhance the perception of moving objects, this paper computes the optical flow vectors between consecutive frames and feeds them, along with each frame image, into a deep learning model to improve the network’s ability to detect and track moving objects. The paper also calculates the displacement and reprojection error between frames.

In NICE-SLAM, a local bundle adjustment method that maintains a list of keyframes is used for pose optimization. However, due to the drift in the hole-filling process, tracking shows instability. Here, optical flow is used for tracking and keyframe selection, following the specific steps below.

To compute the optical flow, we adopt the Lucas–Kanade method [29]. First, ORB feature points (with a threshold of 100 ORB feature points) are extracted from the current frame and its neighboring frames. If the threshold is met, the current frame is added to the keyframe list. Then, nearest neighbor matching is performed to obtain the corresponding optical flow points. To avoid interference in dynamic scenes, feature points within the YOLOv8 detection range are removed. Subsequently, the displacement between frames is calculated as follows:

(20) $$\sum_{i=1}^{n}\left(\nabla I\left(x_{i}\right)\cdot T^{*}\cdot \nabla I{\left(x_{i}\right)}^{⊤}\right)v=-\sum_{i=1}^{n}\left(\nabla I\left(x_{i}\right)\cdot T^{*}\cdot \nabla I{\left(x_{i}\right)}^{⊤}\right)\Delta t$$

where *n* is the number of selected feature points, $\nabla I(x_{i})$ represents the image gradient at feature point $x_{i}$, $T^{*}$ is the pixel transformation matrix, *v* is the optical flow vector, and Δt is the time interval between frames.

The above equation can be expressed in matrix form as a linear system:

(21) Av=b

The optimization objective is shown below:

(22) $$v=\arg\min_{v}{∥Av-b∥}^{2}$$

The estimation of the optical flow vector *v* can be obtained by solving the normal equation:

(23) $$v={\left(A^{⊤}A\right)}^{-1}\cdot A^{⊤}\cdot b$$

The final reprojection error of the feature point is expressed as shown below:

(24) $$\xi_{i}=\nabla I\left(x_{i}\right)\cdot \left(T^{*}v+T^{*}\Delta t\right)$$

Unlike NICE-SLAM, which uses the pixel similarity of keyframes for selection but lacks loop detection, this paper introduces loop detection by evaluating the consistency of optical flow vectors of feature points across three consecutive keyframes in the keyframe list. Keyframes are selected based on their consistency scores. The consistency score is calculated as shown below:

(25) $$\mathrm{score}=e^{-\alpha \;\arccos\left(\frac{v\cdot v_{\mathrm{agg}}}{∥v∥∥v_{\mathrm{agg}}∥}\right)}$$

where α=0.5 and a threshold of score>0.9 is used. If the matching scores of feature points across three consecutive s used. If the matching scores of feature points across three consecutive keyframes exceed this threshold, they are marked as loop keyframes. These loop constraints are then added to the global optimization process in bundle adjustment.

By introducing the above improvements, the system enhances tracking stability in dynamic scenes, effectively incorporates loop detection, and ultimately improves the robustness and accuracy of the SLAM system.

## 4. Experiments

### 4.1. Experiments Settings

**Datasets and Metrics.** We evaluated our system on 3 dynamic datasets. The datasets used for evaluation include TUM RGB-D [30], Kitti [31] and Bonn [32]. The TUM RGB-D dataset provides RGB-D images captured by a Kinect depth camera along with ground truth trajectories obtained through a motion capture system for indoor scenes. This dataset is widely used for the evaluation of RGB-D SLAM algorithms. We selected six high-dynamic sequences and two low-dynamic sequences from the TUM RGB-D dataset for evaluation. The Bonn dataset, collected using an D435i camera, contains numerous dynamic scenes. Compared to the TUM RGB-D dataset, the dynamic scenes in the Bonn dataset are more challenging. We selected eight sequences from the Bonn dataset for evaluation.

To comprehensively evaluate the tracking accuracy, we selected RMSE and STD of Absolute Trajectory Error (ATE) as indicators. We evaluate the speed using frames per second (FPS) and measure memory consumption in terms of GPU usage (in gigabytes, G).

**Baselines.** We compare our tracking and mapping results with state-of-the-art methods, including traditional method ORB-SLAM3 [33], as well as NeRF-based approaches like NICE-SLAM and ESLAM, and other dynamic SLAM methods such as Dyna-SLAM and NID-SLAM [34].

**Implementation Details.** To conduct the experiments, we use PyTorch 1.10 and CUDA 11.8, and we performed the computation on an Intel i7-12700K CPU and a RTX 3090ti GPU equipped with 24 GB of video memory. Specifically, we adopted a weighted photometric loss with a weighting parameter λ = 0.2 on the TUM RGB-D dataset. For each image, we sampled N = 1000 and $N_{t}$ = 200 pixel values, respectively.

### 4.2. Results on TUM RGB-D

Table 1 presents the experimental comparison results on the TUM RGB-D dataset. Overall, ORB-SLAM3 and NICE-SLAM exhibit lower robustness in dynamic scenes. Although ESLAM performs better in certain low-dynamic scenarios, its performance deteriorates significantly in highly dynamic environments. In contrast, the proposed method consistently achieves the lowest ATE RMSE across all dynamic scenes, demonstrating a significant advantage even in complex, highly dynamic settings, and showing strong potential for practical applications.

**Figure 2:**
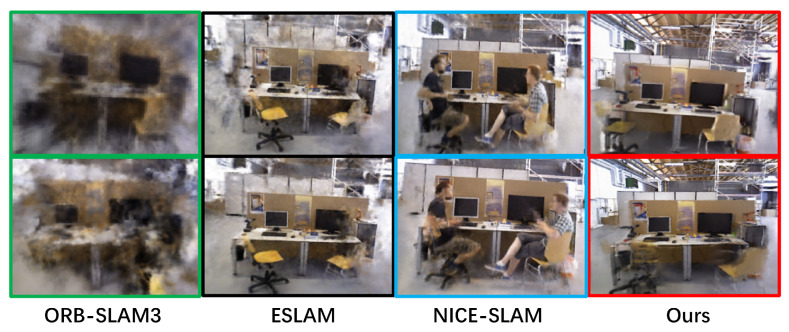
Multi-view rendering results on TUM RGB-D. Our multi-view synthesis results, compared to Orbeez-SLAM, can accurately correct tracking errors and eliminate rendering interference from dynamic human bodies. Although there are still a few dynamic artifacts, these mainly stem from synthesis errors caused by tracking inaccuracies.

Figure 2 demonstrates the multi-view rendering comparison results of our method with other methods on the TUM RGB-D dataset. ORB-SLAM3 and NICE-SLAM fail to remove dynamic objects. Although ESLAM can roughly remove dynamic objects, it leaves behind ghosting artifacts. In contrast, our method achieves clean and artifact-free processing in the regions previously occupied by dynamic objects.

**Figure 3:**
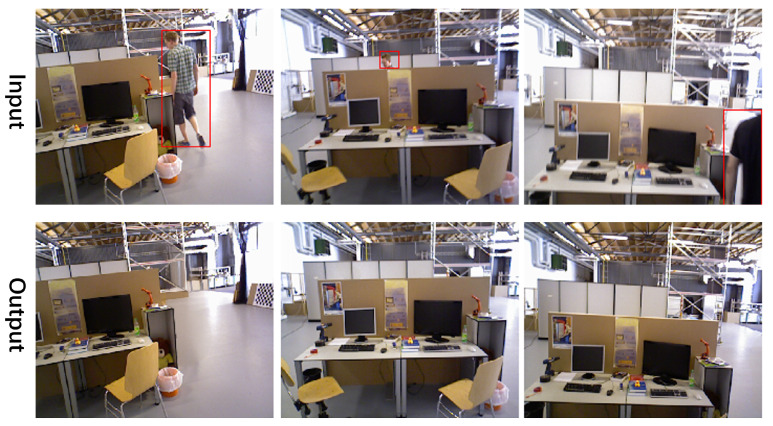
Single-frame rendering results on TUM RGB-D. Our single-frame rendering results on the TUM RGB-D dataset. Compared to the original input, we can stably and accurately eliminate the interference from dynamic human bodies. Moreover, our method is not limited by the size and position of the human body interference, achieving photo-level view synthesis. Additionally, our approach demonstrates robust pose tracking capabilities and, unlike NICE-SLAM, does not experience drift due to hole filling.

Figure 3 shows the single-view rendering results of our method on the TUM RGB-D dataset. The images above are the original pictures, while the images below are the results after rendering. The red boxes highlight dynamic human bodies within the scene. Our method effectively removes human occlusions, achieving scene reconstruction and repair.

### 4.3. Results on Bonn

**Figure 4:**
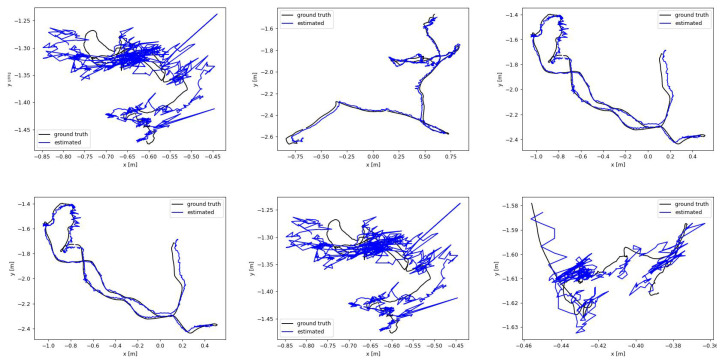
Visualized ATE RMSE (m) results on Bonn. Our method achieves the best tracking results and can robustly recover the pose.

Table 2 presents the experimental comparison results on the Bonn dataset. Due to the multi-target and highly dynamic characteristics of the Bonn dataset, ORB-SLAM3 and NICE-SLAM perform poorly in most scenarios, especially when dealing with dynamic objects, where the errors are larger and they fail to effectively handle these dynamic changes. Although ESLAM performs relatively better in certain scenarios, its errors are still high, particularly in complex scenes, where its accuracy is less satisfactory. In contrast, the method proposed in this paper performs excellently across all test scenarios with the ATE RMSE typically maintained around 3 cm. Figure 4 shows the visualized ATE RMSE results of our method on six sequences from the Bonn dataset. Our method demonstrates its robust tracking capability. Traditional methods typically perform poorly in such challenging scenes, but our approach maintains strong tracking performance.

### 4.4. Results on Kitti

Table 3 presents the experimental comparison results on the KITTI outdoor dataset. Due to the large-scale, high-speed motion, and complex environmental conditions inherent in outdoor scenarios, traditional methods such as ORB-SLAM3, NICE-SLAM, DYNA-SLAM, and NID-SLAM exhibit significant limitations in maintaining robust tracking performance. These methods often perform poorly when handling fast-moving vehicles and long-distance trajectories, resulting in larger errors and occasional tracking failures. For example, although DYNA-SLAM is optimized for dynamic scenes, it struggles to maintain stable performance under high-speed motion and complex lighting conditions. Similarly, while NID-SLAM performs relatively well in certain static scenarios, its accuracy and robustness are significantly affected in frequently changing outdoor environments. ESLAM demonstrates relatively better performance in some scenarios, benefiting from its enhanced feature extraction capabilities. However, in highly dynamic and complex outdoor environments, ESLAM still faces challenges in terms of accuracy and consistency, particularly when dealing with rapid changes in scene structure or lighting conditions. The observed errors under these challenging conditions remain relatively high, impacting the overall reliability of the system.

In contrast, the proposed method exhibits superior performance across all test sequences of the KITTI dataset. Compared to DYNA-SLAM and NID-SLAM, our approach not only performs better in dynamic scenes but also effectively addresses challenges such as lighting variations, occlusions, and long-term trajectory tracking. Our method maintains strong tracking performance even in such demanding environments, highlighting its effectiveness and practical value. This makes it particularly suitable for real-world applications such as autonomous driving and other outdoor robotic systems. Traditional implicit methods, including DYNA-SLAM and NID-SLAM, often struggle to handle the scale and complexity of datasets like KITTI. Our method successfully overcomes these issues through its innovative design and effective handling of dynamic changes, providing a more reliable solution for outdoor SLAM tasks.

### 4.5. Ablation Study

To demonstrate the effectiveness of the proposed method, we conducted ablation study on the balloon1 and balloon2 sequences of the Bonn dataset. As shown in Table 4, the dynamic–static segmentation method significantly improves the system’s localization accuracy. Specifically, in the balloon1 sequence, the ATE of the dynamic–static segmentation method is reduced to 0.026 m, with the STD correspondingly decreased to 0.009 m, indicating that the proposed method effectively handles dynamic objects in the scene. Additionally, Table 5 presents the ablation experiment results for the loop detection method. In the balloon2 sequence, the system with loop detection demonstrated higher accuracy, with the ATE reduced to 0.031 m and the STD reduced to 0.012 m, whereas the system without loop detection showed larger errors. These experimental results further confirm the effectiveness of the proposed method.

We report the computational resource consumption and runtime of different methods in Table 6. Our method shows a significant speed advantage and the lowest GPU memory consumption.

## 5. Conclusions

We propose an SLAM system based on dynamic mask ray correction and sparse feature refinement, which achieves robust tracking and mapping in dynamic environments. By extracting dynamic masks to remove dynamic sampled rays and utilizing static features to construct sparse optical flow and loop closure detection, our method significantly enhances tracking performance and eliminates dynamic interference in mapping. Compared to NICE-SLAM, our approach improves ATE by more than 90%, achieving high-quality mapping with fewer artifacts. Limitations: Our current work relies on depth sensors and a pretraining process, which is primarily aimed at enabling accurate segmentation and ensuring the real-time performance of the system. In future work, we will focus more on monocular sensor-based approaches and leverage large models to reduce the dependence on pretraining, thereby enhancing the generalizability of the system.

## Figures and Tables

**Table 1:** Tracking results on TUM RGB-D. ATE RMSE [m](↓) is used as evaluation metric. The best result is indicated in bold, and the second-best result is indicated with an underline.

Method	ORB-SLAM3	NICE-SLAM	ESLAM	DYNA-SLAM	NID-SLAM	Ours
fr3/w/xyz	0.507	0.305	0.716	0.087	0.091	**0.045**
fr3/w/half	0.254	0.627	0.241	0.065	0.074	**0.036**
fr3/w/static	0.109	0.093	0.036	0.039	0.041	**0.022**
fr3/w/rpy	0.595	0.724	0.189	0.107	0.115	**0.064**
fr3/w/xyz_v	0.764	0.583	0.297	0.078	0.081	**0.043**
fr3/w/half_v	0.351	0.296	0.175	0.059	0.064	**0.034**
fr3/s/xyz	0.013	0.394	0.025	0.053	0.061	**0.021**
fr3/s/half	0.026	0.109	0.019	0.031	0.037	**0.016**

**Table 2:** Tracking results on Bonn. ATE RMSE [m](↓) is used as evaluation metric. The best result is indicated in bold, and the second-best result is indicated with an underline.

Method	ORB-SLAM3	NICE-SLAM	ESLAM	Ours
balloon1	0.078	2.234	0.204	**0.026**
balloon2	0.245	1.989	0.236	**0.031**
move1	0.230	0.213	0.079	**0.023**
move2	0.127	0.816	0.103	**0.027**
crowd1	0.335	1.765	0.317	**0.015**
crowd2	0.762	3.481	1.143	**0.024**
person1	0.723	0.233	0.147	**0.042**
person2	0.971	0.467	0.453	**0.066**

**Table 3:** Tracking results on KITTI dataset. ATE RMSE [m](↓) is used as evaluation metric. The best result is bolded, and the second-best result is underlined.

Method	ORB-SLAM3	NICE-SLAM	ESLAM	DYNA-SLAM	NID-SLAM	Ours
KITTI 00	1.7	7.0	5.9	1.4	4.2	**1.2**
KITTI 01	10.4	47.0	38.6	9.4	28.2	**8.1**
KITTI 02	**5.4**	36.5	23.8	6.7	20.1	5.7
KITTI 03	0.7	5.0	4.4	0.6	1.8	**0.5**
KITTI 04	0.4	2.3	1.8	**0.2**	0.6	0.3

**Table 4:** Ablation study results of the dynamic–static segmentation method. The best result is indicated in bold.

	w/o Dynamic–Static Seg	w/ Dynamic–Static Seg
ATE RMSE (m) ↓	0.078	**0.026**
STD (m) ↓	0.051	**0.009**

**Table 5:** Ablation study results of the loop detection method. The best result is indicated in bold.

	w/o Loop Detection	w/ Loop Detection
ATE RMSE (m) ↓	0.059	**0.031**
STD (m) ↓	0.033	**0.012**

**Table 6:** This table provides a comprehensive evaluation of various methods on the TUM dataset, focusing on key performance metrics such as tracking and mapping speed (measured in milliseconds per frame), frame rate (FPS), and GPU memory usage (in gigabytes). The optimal results for each metric are emphasized in bold, highlighting the best trade-offs in terms of speed and computational resource consumption.

Method	Track. (ms)	Map. (ms)	FPS	GPU Usage
ESLAM	32.6×200	44.3×50	7.5	7.6 G
NICE-SLAM	47.1×200	189.2×60	0.08	14.1 G
DynaSLAM	7.5×20	607.7×50	13.7	8.8 G
Ours	7.2×10	21.0×20	**15.3**	**5.7 G**

## Data Availability

Data are contained within the article.
